# Fetlock Joint Angle Pattern and Range of Motion Quantification Using Two Synchronized Wearable Inertial Sensors per Limb in Sound Horses and Horses with Single Limb Naturally Occurring Lameness

**DOI:** 10.3390/vetsci9090456

**Published:** 2022-08-25

**Authors:** Eleonora Pagliara, Maddalena Marenchino, Laura Antenucci, Mario Costantini, Giacomo Zoppi, Mario Dante Lucio Giacobini, Michela Bullone, Barbara Riccio, Andrea Bertuglia

**Affiliations:** 1 Department of Veterinary Science, University of Turin, 10095 Grugliasco, Italy; 2 Captiks Srl, 00012 Rome, Italy

**Keywords:** kinematics, joint angles, biofeedback, locomotion, fetlock, IMU, variability, range of motion, gait analysis, lameness

## Abstract

**Simple Summary:**

The incidence of disease affecting fetlock joint and associated structures is high in the equine athlete due to the large loads acting on the joint during athletic activity. Therefore, study of the movement of this joint (flexion and extension) is a significant matter of interest. To date, fetlock joint angle pattern and range of motion in horses have been quantified using optical motion capture (OMC). These systems use a number of cameras to film the subject and reconstruct the movement trajectories into a computer model. They are very accurate and precise but require expensive equipment and a laboratory setting. The use of inertial measurement unit (IMU) systems in the field of motion analysis is now widespread. IMU systems use a number of sensors in order to derive the position of the body in the space; they have the advantage of being able to collect kinematic spatio-temporal data when the animal is moving overground with fewer constraints than in a gait lab. To the authors’ best knowledge there are no studies using IMUs sensors in order to record relative fetlock joint angles in horses moving overground. In this study, we wanted first to validate the use of IMUs for kinematic detection of fetlock joint movement on the sagittal axis comparing it to the OMC. Then, we intended to discuss fetlock joint range of motion variability quantified by the IMU system under investigation in lame and sound horses. The IMU system was able to record fetlock joint range of motion just as does the bi-dimensional OMC at walk and trot, in both sound and lame horses. IMU system quantification of fetlock joint range of motion confirmed that the variability was mainly due to lameness in our population of horses. Quantifying joint angle patterns with an IMU system instead of using OMC has the advantage of furnishing real-time bio-feedback of kinematic data. The system can handle various equine gaits and clinical and training conditions outside of expensive laboratory circumstances.

**Abstract:**

Fetlock joint angle (FJA) pattern is a sensitive indicator of lameness. The first aim of this study is to describe a network of inertial measurement units system (IMUs) for quantifying FJA simultaneously in all limbs. The second aim is to evaluate the accuracy of IMUs for quantifying the sagittal plane FJA overground in comparison to bi-dimensional (2-D) optical motion capture (OMC). 14 horses (7 free from lameness and 7 lame) were enrolled and analyzed with both systems at walk and trot on a firm surface. All enrolled horses were instrumented with 8 IMUs (a pair for each limb) positioned at the dorsal aspect of the metacarpal/metatarsal bone and pastern and acquiring data at 200 Hz. Passive markers were glued on the center of rotation of carpus/tarsus, fetlock, and distal interphalangeal joint, and video footages were captured at 60 Hz and digitalized for OMC acquisition. The IMU system accuracy was reported as Root Mean Square Error (RMSE) and Pearson Correlation Coefficient (PCC). The Granger Causality Test (GCT) and the Bland–Altman analysis were computed between the IMUs and OMC patterns to determine the agreement between the two systems. The proposed IMU system was able to provide FJAs in all limbs using a patented method for sensor calibration and related algorithms. Fetlock joint range of motion (FJROM) variability of three consecutive strides was analyzed in the population through 3-way ANOVA. FJA patterns quantified by IMUs demonstrated high accuracy at the walk (RMSE 8.23° ± 3.74°; PCC 0.95 ± 0.03) and trot (RMSE 9.44° ± 3.96°; PCC 0.96 ± 0.02) on both sound (RMSE 7.91° ± 3.19°; PCC 0.97 ± 0.03) and lame horses (RMSE 9.78° ± 4.33°; PCC 0.95 ± 0.03). The two systems’ measurements agreed (mean bias around 0) and produced patterns that were in temporal agreement in 97.33% of the cases (*p* < 0.01). The main source of variability between left and right FJROM in the population was the presence of lameness (*p* < 0.0001) and accounted for 28.46% of this total variation. IMUs system accurately quantified sagittal plane FJA at walk and trot in both sound and lame horses.

## 1. Introduction

Equine fetlock is the rotary joint which experiences the largest load during locomotion, irrespectively of the gait [1]. Functionally, the fetlock joint is part of the equine limb elastic suspensory apparatus whose biomechanical behavior is explained by the distal spring-mass model [2]. Based on this, horses can run in an almost passive manner, thanks to the elastic energy storage properties of the suspensory ligament and digital flexor tendons [1,2,3]. Movement in the sagittal plane predominates in the fetlock joint and consists in flexion (during most of the swing phase of the stride) and extension (during the end of the swing phase and the stance phase) [4]. The extension of the joint determines the load on associated tendons and ligaments and, as a consequence, the incidence of disorders affecting the fetlock is reported to be high in the equine athletes [5,6,7].

Dynamic changes of sagittal fetlock joint angle (FJA) during locomotion, also referred to as fetlock joint angle pattern (FJAP), have been described at walk and trot in sound horses and in horses with experimentally induced forelimb lameness [8,9]. Of note, these observations have not been validated in a cohort of horses with naturally occurring lameness to date. All currently available data on FJAP have been acquired/obtained by means of 2-D and 3-D optical motion capture (OMC) technology, whose application requires highly standardized laboratory settings and is limited by space constraints, lighting conditions, and the possibility to perform only specific tests [10] There is growing interest in portable devices that can be used as OMC surrogates for gait assessment/analysis, in both research and clinical settings, and new scientific evidence on this subject is warranted. 

Inertial sensors are devices characterized by small size and limited cost where an accelerometer or a gyroscope are used to transduce the orientation and the inclination of a body segment into measurable spatio-temporal kinematic signals at high sampling rates (~200 Hz) [11]. Wearable inertial sensor measurement units (IMUs) allow wireless data transmission with the advantage of bypassing high costs and technical limitations associated with OMC technology. For these reasons, they have been largely employed in equine gait analysis and, more recently, as an aid in equine lameness detection in the field [11,12]. IMU systems (IMUs) have been placed on the distal extremity to assess distal limb displacement [13], translation of metacarpus as a rigid segment [14], protraction and retraction angles [15], as well as for temporal and spatial variables detection of the strides during different gaits [16,17,18]. To the authors’ knowledge, there are no reported data investigating FJAP using IMUs in horses.

The present work was undertaken under the hypothesis that equine gait analysis data concerning FJAP acquired with commercially available IMUs are comparable to those obtained with 2-D OMC. Based on the current literature, and on the possible relevance of FJAP asymmetry in equine lameness, we also hypothesized that FJAP differed in sound horses vs. horses with naturally occurring lameness. To pursue these aims, we have described and compared FJAP data from 7 sound and 7 lame horses, contemporarily acquired using a wireless 8-sensor IMU-system and with a 2-D OMC.

## 2. Material and Methods

### 2.1. Study Design

In this prospective observational study, fourteen horses were enrolled with a 1:1 ratio between groups (sound vs. lame horses). Enrollment started in January 2021 and ended in November 2021. The sound horses were recruited from a jumping training center affiliated with our institution. The lame horses were recruited among cases referred to the Equine Clinical Service at the Veterinary Teaching Hospital of University of Turin. A written informed consent was obtained by all owners of the horses. Ethical approval of the study protocol was obtained by the local competent committee (protocol n. 2796/2020; approval date 15 December 2020).

Horses were assigned to the sound group after subjective assessment by two experienced clinicians (ACVSMR diplomates) and consensual agreement on the absence of lameness or any relevant gait alterations secondary to neurological or non-orthopaedic disorders. To be included in the sound group, horses had to be in training and free of orthopaedic disease for at least 2 years. We deliberately chose sound horses with similar physical conformation (withers height-weight) to reduce variability in FJAP [19]. Lame horses were included in the study if they presented a moderate to severe single limb lameness ranging from grade 3 to 4 based on the AAEP scale scoring system [20] due to an orthopaedic disease. The diagnosis in each horse was confirmed based on a combination of a subjective examination of lameness, results of intra-articular or perineural nerve blocks, and diagnostic imaging.

Each horse enrolled in the study underwent a standardized locomotion test during which FJAPs were simultaneously evaluated in all 4 limbs with the two systems tested (IMU and OMC) at walk and trot as detailed below.

### 2.2. IMU System

**Figure 1:**
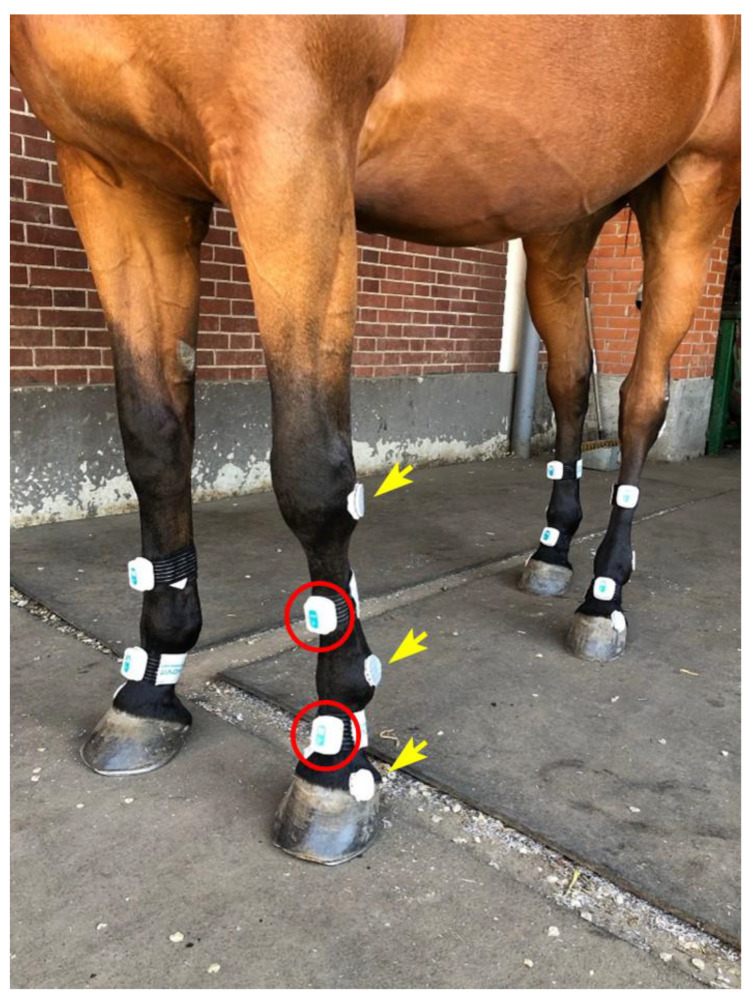
Placement of IMUs (red circle, identified only in one limb) and optical markers (yellow arrows, OMC technology) on equine distal limbs for data acquisition. The IMUs were fixed to the dorsal metacarpus/metatarsus and to the dorsal aspect of the pastern in the four limbs by means of plastic docking bases attached to elastic bands. Optical markers were glued to the skin on the lateral aspect of the joints studied.

The inertial system used (MOVIT System G1, Captiks SRL Rome- http://www.captiks.com/it/prodotti/movit-system-g1-3d (accessed on 28 June 2022) consists of 8 wireless inertial sensors measuring 48 × 39 × 18 mm and weighing 40 g each. Sensors are made of a triaxial acceletometer (full-scale range of ±2 g) and a triaxial gyroscope measuring angular velocity in the range of ±2000°/s, with a sampling rate of 200 Hz. By using the quaternion (6 degrees of freedom), sensors measure changes in orientation of the limb to which it was applied. In detail, sensors detect changes in rotation along the sagittal axis (backward/forward and rolling, respectively), vertical axis (up/down and yawing), and transverse axis (right/left and pitching). The system also features a patented calibration that allows the sensors to have the same global reference system, via three orthogonal positions assumed by a baseplate (U.S. Patent 102016000041519, 14 November 2018). Starting from the same global reference system, it is possible to consider two sensors and measure relative orientation data. In our case, relative orientation data yielded information on the dorsal fetlock joint angles by tracking movements of the pastern relative to the metacarpus or metatarsus. The system is provided with a calibration docking station that can lodge contemporarily the 8 sensors used. Each sensor has a docking base which makes positioning of the sensors over the body easy. Each docking base is indeed provided with velcro, which allows the adhesion on an elastic band provided by the manufacturer (Captiks S.r.l., Rome, Italy). In our study, the elastic bands were fixed around each distal metacarpal/metatarsal bone and pastern, with the sensor positioned on the dorsal aspect of the limb (Figure 1). Each sensor is provided with an internal flash memory card for data storage. Although the system also allows real time data recording with a dedicated software (Motion Studio, Rome, Italy) , in this case the sampling rate is lower (100 Hz) than acquiring data into the internal storage (200 Hz). To obtain the maximum sampling rate and to avoid data loss due to wireless communication we opted for internal storage.

Before each test, IMUs were calibrated following the steps described in the U.S. Patent 102016000041519, Nov.14, 2018. The steps are as follows: perform two 90° rotation with the calibration base holding the 8 sensors, put the 8 sensors and the optical markers on the 4 limbs of the horse (Figure 1), maintain a static position of all couples of IMU (pastern-cannon bone) for few seconds with the horse standing with the limbs squarely positioned to finish the calibration phase. By doing this, the system can now measure the relative angle of the fetlock joint (dorsal FJA) as the outcome unit. Conventionally, FJAs were set at 0° during calibration. Thus, positive and negative sagittal FJA indicate flexion and extension of the joint, respectively, compared to the initial calibration point. 

The Motion Studio software provided by the IMU sensor manufacturer allows starting and ending data acquisition, as well as data visualization in real time [21]. 

### 2.3. OMC Technology

**Figure 2:**
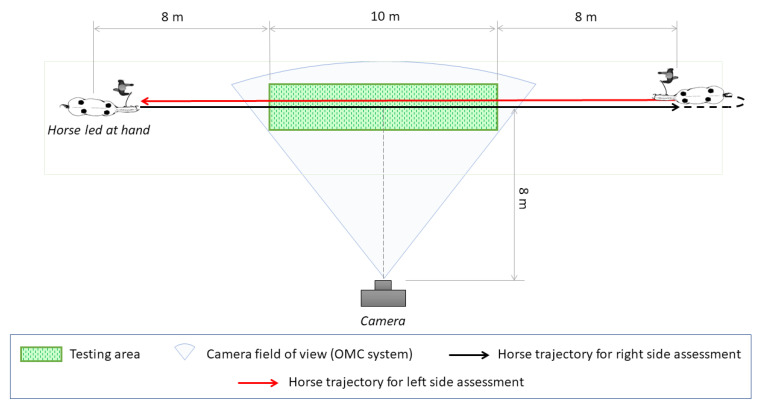
Schematic representation of the set up used for the standardized locomotion test performed.

Optical data were obtained in a standardized environment (testing area). A digitalcamera (Exilim EX-FH20, Casio, Tokyo, Japan, full HD camera resolution 1920 width × 1080 height) with a recording time resolution of 60 Hz was used, placed at a distance of approximately 8 m from the center of the testing area through its transverse axis. The position of the camera was chosen to include the whole testing area (10 m long) within a single field of view (Figure 2). This was based on what is currently considered the minimal data requirement for equine gait analysis, corresponding to three complete and consecutive strides [22]. In more detail, as the average speed of the horse during trot is 3 m/s and average stride length is 250 cm [23], we calculated the distance required for capturing a minimum of 3 strides at trot at constant speed and we choose 10 m as the optimal length of our testing area. The objective of the camera was positioned perpendicular to the long axis of the testing area. For OMC analyses, three flat, circular retroreflective passive markers measuring 40 mm in diameter were glued on the lateral aspect of all limbs at the level of the center of rotation of carpus, tarsus, fetlock, and distal interphalangeal joints.

Video recordings were digitized with a dedicated software for gait analysis (Bio movie, Infolab media, Aosta, Italy). The fetlock joint angles of all limbs were computed at 60 Hz from reflective marker trajectories, by means of a dedicated software tool, and exported in an Excel file. Fetlock joint angles were expressed as the dorsal angle of the pastern axis relative to the vertical (0°) angle of the metacarpus/metatarsus axis. In this way, fetlock entension produced a decrease in FJA while fetlock flexion produced an increase in FJA, and patterns were more easily comparable to those obtained with the IMU system. 

### 2.4. Standardized Locomotion Test

For the standardized locomotion test performed, horses were first walked and then trotted (if allowed by their pathological condition) back and forth led by hand, with the animal’s handler at the opposite side of the camera (Figure 2). The standardized locomotion test was performed on a firm surface (concrete). The surface was the same for all the trials, in a defined testing area, consisting of a 10 × 2 m rectangle within a dedicated trotting-up area (Figure 2). Horses walked or trotted in line starting approximately 8 m before the testing area at both sides to obtain a constant speed and maintained this throughout the test. 

### 2.5. Data Acquisition

Data acquisition with the IMUs was started manually before the horse entered into the testing area and was stopped manually when the horse exited the area.

Data acquisition for OMC was performed as described above. Videorecording was focused on the pair of limbs on the side of the camera (right fore [RF] and right hind [RH] while walking and trotting forth, and left fore [LF] and left hind [LH] while walking and trotting back), and lasted as long as possible from the moment the horse entered in the field of view of the camera (testing area) to when it went out of view. A trial was rejected if the horse was judged by the observer as moving inconsistently or not freely within the testing area. 

Both systems generated Excel files (one per limb per test) with FJA measurements over time.

### 2.6. Data Processing

Fetlock joint angles data derived from the two systems were imported in MATLAB (version 2020R, The MathWorks Inc., Natick, MA, USA). A custom-written specific script was designed to compare FJAP (Supplementary Materials). Briefly, resampling of the video signal (from 60 to 200 Hz) was performed using MATLAB’s spline interpolation function in order to have both sets of data at 200 Hz. Both signals were low-pass filtered with a 4-order Butterworth filter with a cutoff frequency of 10 Hz. The data obtained from the two systems were synchronized at the point of absolute minimum between the first two maxima of the two signals. Absolute FJAP obtained with the two systems were also aligned on the y axis (°) by subtracting the difference of the two patterns calculated at the synchronization point. 

In line with previous work [24], stride segmentation and detection of toe-on and toe-off events was performed based of resultant angular velocity data recorded from the gyroscope in the IMU sensor mounted on the pastern of the limb studied.

**Figure 3:**
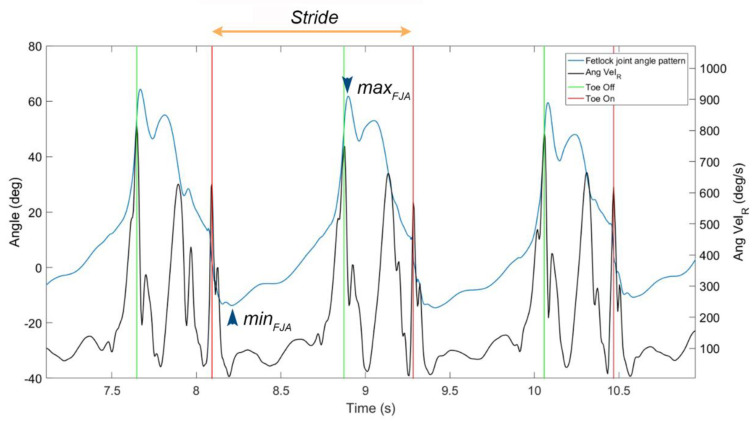
Results of stride segmentation by means of the gyroscope signal quantified at the pastern and identification of minimal and maximal FJA during the stride used for calculation of fetlock joint range of motion (FJROM). Data from a lame horse at walking speed on asphalt. Ang vel_R_: resultant angular veolcty; min_FJA_: minimal FJA within the stride; max_FJA_: maximal FJA within the stride.

After stride segmentation, the fetlock joint range of motion (FJROM) was computed from IMU system data as the difference between the maximal and minimal FJA recorded within the same stride. Mathematically, this was calculated as: FJROM_stride_ = absolute value [max(FJA_stride_) − min (FJA_stride_)] (Figure 3). FJROM was calculated as the mean of ROM of three strides for every limb.

### 2.7. Statistical Analysis

The Pearson correlation coefficient (PCC) and root mean square error (RMSE) between the FJAs detected with the two systems under investigation at each time point (200 Hz) were calculated using the MATLAB corrcoef function (PCC) and the following formula (RMSE) for each fetlock joint studied (RF-RH-LF-LH) and for each gait (walk-trot):

(1) $$\mathrm{RMSE}=\sqrt{\frac{\sum_{i=1}^{n}{\left(x_{i}-y_{i}\right)}^{2}}{n}}$$

PCC was used to measure the strength of the linear relationship between the two patterns and RMSE (°) to measure the difference between FJA values detected with IMUs and OMC for each joint at each gait. The mean values of PCC, RMSE, and their standard deviation were calculated at walk and trot in sound and lame horses using Microsoft Excel (Version 16.58, 2013, Microsoft Corporation, Redmond, WA, USA).

To calculate the agreement between the two systems, Bland–Altman analyses were run separately on data of single FJA values (200 FJA/second) and FJROM (mean of values calculated from 3 strides) measured by the two systems, accounting for repeated measures [25]. Data obtained at walk and trot were analyzed separately. The Granger causality test (α = 0.01) was also computed to predict temporal agreement between IMUs patterns and 2-D OMC patterns in sound and lame horses. 

The coefficient of variation (CV [%] = s d/mean × 100) of the FJROM detected by IMU between right and left forelimbs and between right and left hindlimbs were calculated for all animals enrolled in the study over 3 consecutive strides, for each considered gait (walk and trot). In lame horses, only the lame limb and its contralateral were studied with this aim. 

The effect of gait (walk/trot), lameness (sound/lame), and pair of limbs (front-hind) on CVs was assessed by means of a three-way ANOVA (α = 0.05) after verifying normal distributin of the data with the Shapiro–Wilk normality test. The model did not consider the horse as a clustering or independent variable. 

## 3. Results

### 3.1. Animals

The sound horses were all Warmbloods (3 mares and 4 geldings), all used for show jumping, aged 10.8 ± 3.5 years (mean ± SD; range: 7–18 years). Their weight was 509 ± 45 kg (range: 450–580 kg) and withers height 163 ± 9 cm (range: 153–175 cm). All sound subjects had no history of lameness in the previous 2 years and were trained regularly at the time of the study. Lame horses were of different breeds (details reported in Table 1). They were 4 mares and 3 geldings, 2 used for leisure activity, 2 for breeding purposes, and 3 for show jumping. Their mean age was 12.3 ± 6.6 years (range: 4–23 years), weight 514 ± 70.91 kg (range: 410–600 kg) and height at the withers 158 ± 10 cm (range: 145–175 cm). All horses studied were shoed at the time of the test. 

### 3.2. Standardized Locomotion Test

The horses did not show any sign of stress or discomfort due to the presence of IMUs fixed to their distal limbs and were judged to move freely after a brief initial adaptation period. The average walking speed was 1.53 ± 0.19 and 1.51 ± 0.35 m/s in sound and lame horses, respectively. The average trotting speed was 3.56 ± 0.78 and 3.92 ± 0.85 m/s in sound and lame horses, respectively. Horse z1 was not tested at trot because it was too lame to trot at constant velocity.

#### 3.2.1. Sagittal FJA Curves Generated by MOVIT IMU System

**Figure 4:**
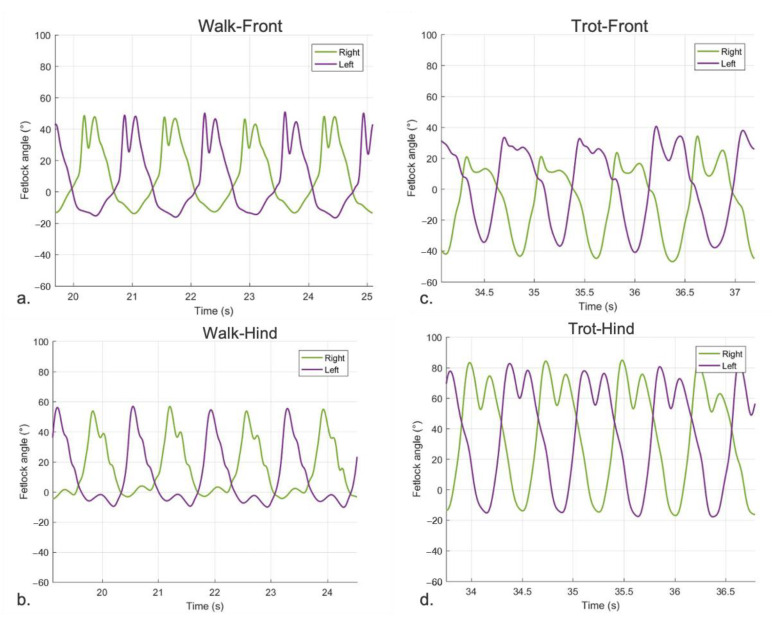
Fetlock joint angle pattern quantified by the MOVIT IMU system in a sound horse over time. Positive values of angles (°) represent flexion and negative values extension of the fetlock joint. (**a**) left front (violet-mean 57.84°) and right front (green-mean 56.72°) at walk; (**b**) left hind (violet-mean 67.01°) and right hind (green-mean 63.55°) at walk; (**c**) left front (violet-mean 78.92°) and right front (green-mean 78.05°) at trot; (**d**) left hind (violet-mean 94.14°) and right hind (green-mean 92.03°) at trot.

In sound horses, the pattern describing sagittal FJA at walk showed a symmetric double peak of flexion and two extension peaks (Figure 4a,b). At trot, the curve was characterized by a symmetric double peak of flexion and a single peak of extension for each stride (Figure 4c,d). The shape and the amplitude of the FJA curves were similar between pairs of limbs (left vs. right forelimbs and left vs. right hindlimbs; Figure 4).

**Figure 5:**
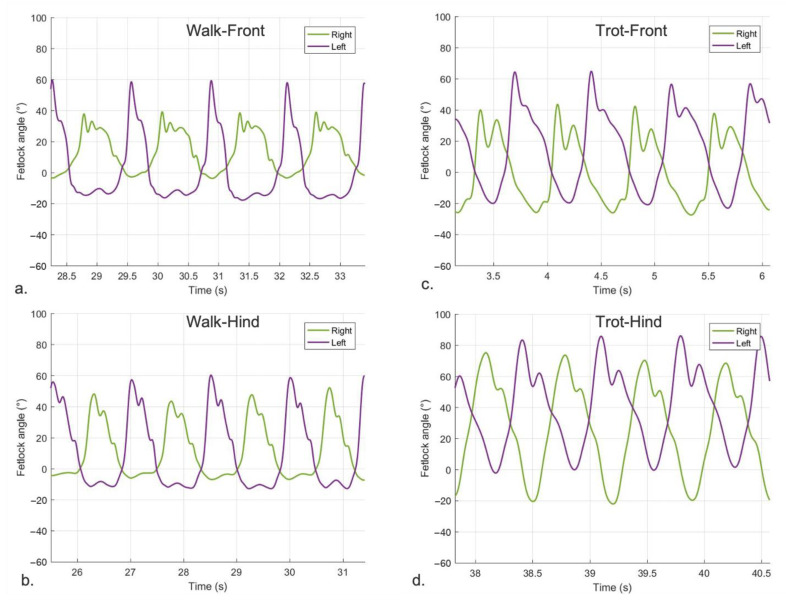
Panels show fetlock joint angle pattern quantified by the MOVIT IMU system in the lame limb and its contra-lateral over time in 4 different horses. Positive values of angles (°) represent flexion and negative values extension of the fetlock joint; (**a**) horse z01-right front lame (green-mean 44.22°) and left front contralateral (violet-mean 75.60°) at the walk; (**b**) horse z05-right hind lame (green-mean 52.88°) and left hind contralateral (violet-mean 70.38°) at walk; (**c**) horse z08-right front lame (green-mean 87.98°) and left front contralateral (violet-mean 98.69°) at trot; (**d**) horse z06-left hind lame (violet-mean 73.35°) and right hind contralateral (green-mean ROM 94.34°) at trot.

In lame horses, the curves describing FJA at walk and trot in the lame limb were characterized by an asymmetric pair-to-pair double peak of flexion for each stride and an asymmetric pair-to-pair single peak of extension at each stride at the trot. The shape and the amplitude of the patterns were different between the lame limb and its contra-lateral, with the lame limb showing a constant decrease in amplitude resulting in a smaller ROM compared to the contra-lateral. This pattern was similar either at walk (Figure 5a,b) or trot (Figure 5c,d). 

In sound horses, the mean FJROM value in front limbs captured by the MOVIT IMU system was 57.79° ± 4.50° at walk and 82.83° ± 6.64° at trot. The mean FJROM in hind-limbs at walk was 60.48° ± 6.10°, and 89.40° ± 4.89° at trot. The FJROM coefficient of variation (CV) between pairs of front limbs (left vs. right) was 3% at the walk and 2.51% at the trot. The CV between paired hind limbs (left vs. right) was 3.91% at the walk and 3.60% at the trot. The mean degree of difference in FJROM between paired front limbs was 1.76° at walk and 0.57° at trot, and between hind limbs was 0.86° at walk and 1.58° at trot. 

**Figure 6:**
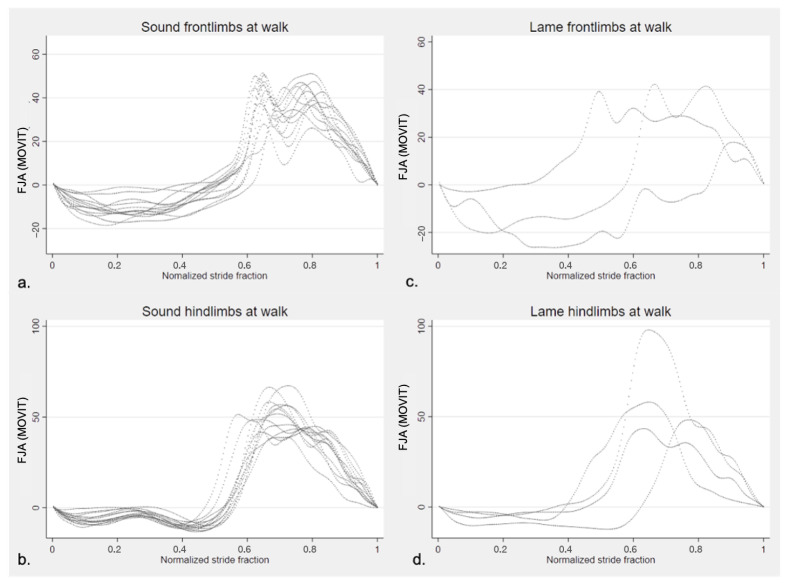
Fetlock joint angle pattern of one normalized entire stride of the horses in the study quantified by MOVIT IMU system at walk. Positive values of angles (°) represent flexion and negative values extension of the fetlock joint. (**a**) left and right sound forelimbs (*n* = 14); (**b**) left and right sound hindlimbs (*n* = 14); (**c**) lame forelimbs (*n* = 3); (**d**) lame hindlimbs (*n* = 4).

**Figure 7:**
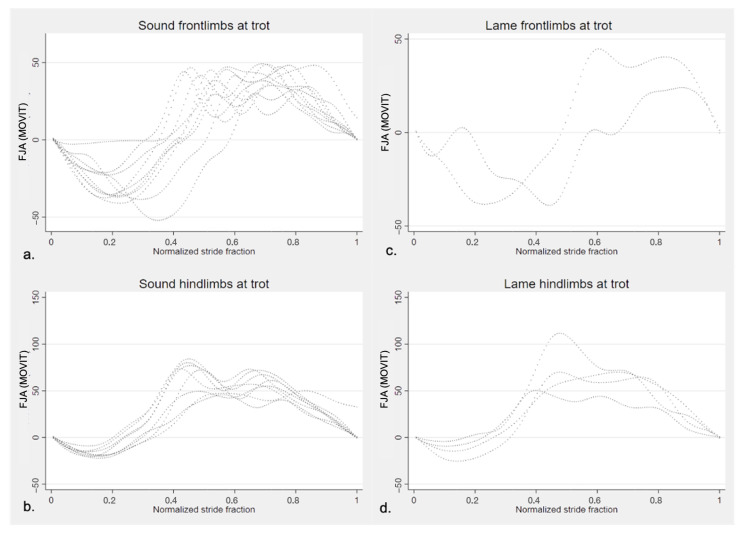
Fetlock joint angle pattern of one entire stride (normalized) of the horses in the study quantified by MOVIT IMU system at trot. Positive values of angles (°) represent flexion and negative values extension of the fetlock joint. (**a**) left and right sound forelimbs (*n* = 14); (**b**) left and right sound hindlimbs (*n* = 14); (**c**) lame forelimbs (*n* = 2); (**d**) lame hindlimbs (*n* = 4).

In lame horses, the CV of FJROM between the lame limb and the contralateral was 15.47% at the walk and 7.06% at the trot. The mean degree of difference in the FJROM between the lame limb and contralateral was 19.84° in lame at the walk and 8.44° when considering all lame horses at two gaits. The normalized stride fraction of the FJA in sound and lame limbs of the horses present in the study are illustrated in Figure 6 (walk) and Figure 7 (trot).

#### 3.2.2. Determinants of FJROM Variability in MOVIT IMU System-Acquired Data

**Figure 8:**
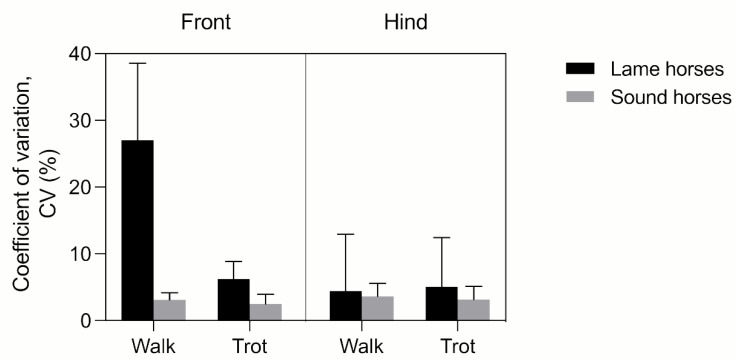
Effect of lameness, gait, and front vs. hindlimbs on CV between left and right limb of the horses in the study.

The CVs of FJROM of consecutive strides (up to three) of the same limb were consistently < 5%, irrespective of gait, front vs. hindlimbs, and the presence of lameness. The CV between right and left FJROM was significantly affected by lameness (*p* < 0.0001), gait (*p* = 0.0029), and front vs. hindlimbs (*p* = 0.0233). Left to right CV FJROM differences in lame and sound horses are shown in Figure 8.

The presence of lameness per se, as most significant, accounted for 28.46% of total variation to explain the left to right FJROM difference. The gait accounted for 10.44%, whereas the presence of front vs. hind limb lameness accounted for only 5.7% of the total variation.

#### 3.2.3. Agreement between MOVIT IMU and 2-D OMC Technology for FJA Quantification

**Figure 9:**
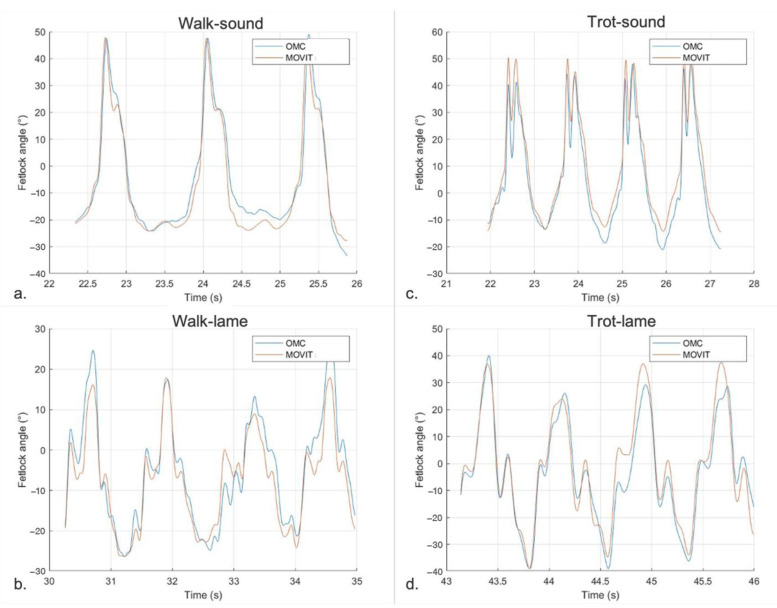
Pattern of FJA over time measured by the MOVIT IMU system (MOVIT-orange) and the 2-D OMC (OMC-blue) in four selected cases. (**a**) Left hind at walk in a sound horse (RMSE = 7.09°—PCC = 0.96). (**b**) Left front at walk in a lame horse (z2-RMSE = 4.69°—PCC = 0.95). (**c**) Right front at trot in a sound horse (RMSE = 7.47°—PCC = 0.97). (**d**) Left front at trot in a lame horse (z2-RMSE = 5.91°—PCC = 0.96).

In sound horses, RMSE between the two methods was 7.77 ° ± 3.42° at walk and 8.06° ± 2.99° at trot, while PCC was 0.96 ± 0.03 (*p* < 0.001) at walk and 0.97 ± 0.02 (*p* < 0.001) at trot. According to the Granger causality test, the pattern of the two systems studied was in temporal agreement in 54/56 cases at walk (94.7%) and in 55/56 cases at trot (*p* < 0.01) (Figure 9a,c).

In lame horses, RMSE between the two systems was 8.68° ± 4.03 at walk and 10.06° ± 4.39 at trot, while PCC was 0.95 ± 0.04 (*p* < 0.001) at walk and 0.96 ± 0.03 (*p* < 0.001) at trot. According to the Granger causality test, the pattern of the two systems studied was in temporal agreement in 55/56 cases at both walk and trot (*p* < 0.01) (Figure 9b,d). 

**Figure 10:**
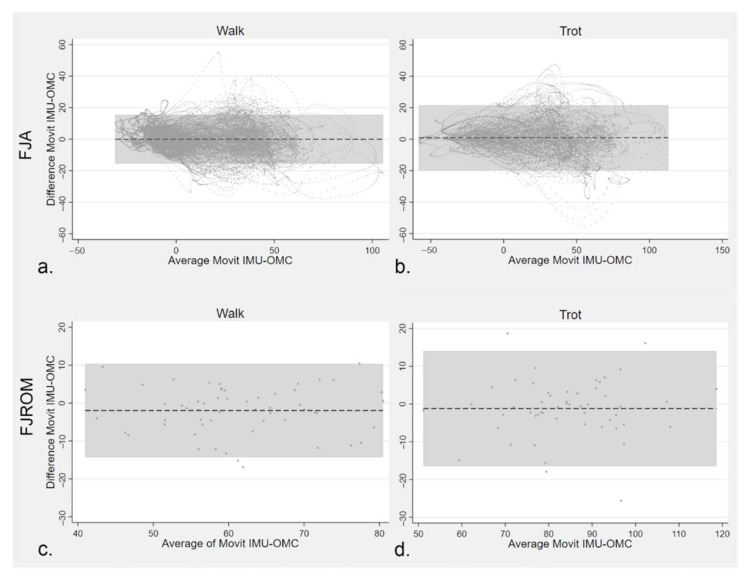
Bland–Altman plots indicating agreement between MOVIT IMU system and 2-D OMC concerning FJA (**a**,**b**) sampled at 200 HZ and derived FJROM (**c**,**d**). Data were obtained at walk ((**a**,**c**) respectively with *n* = 389,377 and *n* = 14) and at trot ((**b**,**d**) respectively with *n* = 16,333 and *n* = 13). Dashed horizontal lines show the mean differences between measures taken with the IMU and OMC technology. The grey areas indicate a 95% CI of the differences.

According to Bland–Altman analyses, the MOVIT IMU system and the 2-D OMC measurements quantifying FJA in both lame and sound horses agreed at both walk (mean difference = −0.09; limits of agreement = −15.60, 15.41) and trot (mean difference = 0.31; limits of agreement = −20.58, 21.21). The agreement of FJROM assessed with Bland–Altman analysis revealed a reduction of mean bias between the two systems tested at both walk (mean difference= −1.96; limits of agreement= −14.26, 10.34) and trot (mean difference= −1.19; limits of agreement= −16.45, 14.08), as shown in Figure 10. 

## 4. Discussion

The aim of this research was to describe, for the first time, equine FJA curves as detected by the MOVIT IMU system in field conditions at the walk and trot. Curves were obtained from sound horses and from horses with naturally occurring lameness localized to a single limb. The curves obtained with the MOVIT IMU system were also compared with those obtained with a 2-D OMC technology. Shape and amplitude of the curves obtained with the two systems agreed and were comparable. 

The study also demonstrated that the variability of fetlock joint range of motion registered with IMUs in lame horses moving in a straight line was mainly related to the presence of lameness. 

### 4.1. Equine FJA Curves in Sound and Lame Horses

Horses with induced forelimb lameness showed altered fetlock joint extension patterns compared to sound horses, detectable at walk and trot [8,9,26]. Sound horses show a similar degree of fetlock extension in lateral pairs of limbs (right and left forelimbs or hindlimbs). In lame horses, fetlock extension is reduced in the lame limb while increased in the contralateral limb [26]. Of note, all available work concerning the effect of lameness on fetlock extension and, more broadly, on FJA, employed experimentally induced forelimb lameness models, in the absence of validation in spontaneously occurring lameness. Our data confirm that the shape and amplitude of FJA patterns differ in sound and natural occurring lame horses, irrespective of the anatomical localization of the lameness. 

In sound horses, FJROM variability calculated over 3 strides of the same limb was approximatively 3% at both walk and trot for both front and hindlimbs, in line with the work of Degueurce [19]. Considering that absolute gait symmetry is unnatural and that some horses are characterized by natural gait laterality [27], we considered 3% as very low. Although maximal joint extension and flexion were repeatable between strides of the same animal for the same limb, the observed FJA pattern in the lame limb was asymmetric compared to the contralateral sound limb in our study. The variability of FJORM was higher in lame (11%) compared to sound horses (3%), and at walk (15%) compared to trot (7%). The almost halved variability at trot in the group of lame horses could be a spurious result but was considered mainly linked to the fact that the FJROM is greater at trot compared to walk and symmetry and regularity increase with velocity [28]. FJROM variability at trot was higher in the lame group compared to the sound (7%–3%) but, although not standardized in our study, average velocity at trot was similar in both groups. So, as velocity could have accounted for the same variability between the groups, the difference in FJROM variability between lame and contra-lateral limb could be considered relevant. 

In our study, the inclusion criteria were only based on the severity of lameness, so animals of different breeds were inserted in the lame group. This could have played a consistent role in the variability of kinematic parameters due to different motion patterns in the fetlock joints in terms of animation of the movement and elasticity between animals. However, it has been demonstrated that the effect of height at the withers is minimal when considering angular parameters and does not affect temporal parameters of the stride [29]. In the work of Ishihara et al. [30], a larger coefficient of variation for kinematics variables and larger variation from the optimal motion pattern have been present in horses showing more severe lameness, reflecting the findings of our study where horses with severe lameness were included. 

### 4.2. Agreement between the Systems Studied

The precision of the MOVIT IMU system in detecting FJA in sound and lame horses was considered good, as according to Cuesta et al. [31]. RMSE between the two methods ranged between 5° to 10° at both walk and trot in lame and sound horses (mean RMSE = 8.83°). According to Poitras et al. [32], the values of PCC showed an excellent correlation between the IMU system and 2-D OMC as it was over 0.9 at two gaits and in the two groups (mean PCC = 0.95). 

Based on the Bland–Altman analysis, the mean bias between the two systems under study was negligible, when both FJA and FJROM were used as input data. Limits of agreement were narrower for FJROM compared to FJA data. For FJA, the 95% CIs indicated that bias could range from 20° to −20° which prompted further data inspection and analysis. The following observations were made, which could explain our result: 1. patterns were not perfectly aligned, which produced the largest differences when the slope of the curve was proximal to the vertical; 2. larger differences were observed for higher positive averages corresponding to the swing phase of the stride during fetlock joint flexion. A limited marker movement could certainly happen during fast oscillating events and marker displacement is a possible source of error for cranio-caudal estimates [13]. In addition, acquiring videos at 60 Hz makes the comparison with a 200 Hz system less reliable. For FJROM, the bias between the systems ranged between 10° and 16° degrees, which was considered acceptable in relation to the large variation in angle degree values and the nature of the experimental settings. Farber et al. [33] found a 10%−15% error in flexion extension assessment of dorsal spinous processes due to skin displacement of reflective markers. That said, the agreement was judged satisfying and the reported bias error was overestimated.

Although the MOVIT IMU system could retrieve angles in all three dimensions, only data concerning the sagittal dimension have been taken into consideration for the purpose of this study as the comparison has been made with the 2-D OMC technology. However, movement in the sagittal plane is the predominant type of motion of the fetlock joint [34] and the variability between sound horses in fetlock joint movement in the sagittal plane has been reported to be low [19]. 

The previous study on fetlock joint kinematics in the horse has been reported using 2-D OMC technology and 3-D OMC on hard surfaces at walk [35,36] and trot [37] with horses exercising on the treadmill [38]. Values of FJROM measured with the MOVIT IMU system in our study are in line with previous results of sagittal plane kinematics for a walk [39] and trot [37,40,41] in sound horses. More precisely, the mean FJROM of forelimbs at walk quantified by the MOVIT IMU system was 58.56° ± 4.41°, in comparison to 62° ± 1.4° in the work of Back et al. [41] with 2-D OMC and 62° ± 7° in the work by Clayton et al. [37] by 3-D OMC. At the trot, the mean FJROM of forelimbs measured by the MOVIT IMU system was 80.66° ± 5.86°, in comparison to 80.6° ± 7.1° and 77° ± 5° in the previously cited works, respectively.

### 4.3. Pros of IMU System Recording FJA

The portability of the MOVIT IMU system (due to its internal memory storage) could enable the possibility of its use to study limb kinematics in vivo during different types of clinical conditions and gait velocity encountered in practice as demonstrated by the findings of this article. One of the main limitations of OMC technology is, in fact, the limited camera field of view. Tridimensional OMC requires a high number of cameras and supporting infrastructure, so has mostly been limited to a fixed laboratory environment. 

To the authors’ best knowledge, two commercial systems rely on extremity-mounted IMUs that detect angular parameters in the sagittal plane (EquiMoves-Pegasus). The EquiMoves system relies on a single sensor positioned on a bone segment (metacarpal/metatarsal bone) and it could only measure the orientation of the cannon bone as a rigid element (absolute angle). In our study, the position of two IMUs over the fetlock joint has allowed the quantification of the relative angular pattern of the anatomical joint. This advantage permits, in addition to a complete and realistic spatial study of the kinematics of this joint, a detailed quantification of temporal variables (stride, stance, and swing duration). The IMU system under investigation could register and quantify the ROM for all individual limbs enabling a complete comprehension of limb motion that could be useful to objectively assess performance, symmetry of movement, clinical improvement, and efficacy of rehabilitation protocols.

### 4.4. Limitations

This study has some limitations, first of all, the use of 2-D instead of 3-D OMC for validation purpose. The studies on the validation of the EquiMoves and Pegasus systems were realized on a treadmill [11,14] and reported an overall accuracy between the systems higher than ours. To compare the IMU system under investigation, we used a 2-D OMC technology with a lower sampling rate (60 Hz) than those reported in the previously mentioned study, so this method is suboptimal for consideration as a gold standard. Since this IMU system is designed for everyday use by practitioners working-up clinical cases, we decided to perform an experiment that best matched this condition, precluding any laboratory setting. The accuracy of the IMU system has not been assessed on different surfaces. 

## 5. Conclusions

The IMU technology can be used to assess FJA, approving its use in gait and performance analysis. The system presented in this study provides real-time wireless FJA patterns detection, with the potential for real-time biofeedback to the user. The proposed IMU system registers FJROM for all individual limbs simultaneously in a horse, and this could enable better understanding of the more extensively affected limb and compensatory patterns during lameness. To the authors’ best knowledge, this is the first study in which IMUs are applied over fetlock joints to record relative angle pattern overground. Its use in the field of objective lameness evaluation is under investigation and further studies aimed at quantifying biologically significant differences in FJROM, FJA patterns and limb phasing related to lameness are still needed.

## Figures and Tables

**Table 1:** Signalment, source, and severity of lameness in horses belonging to the lame group.

Horse	Age	Breed	Pathology	Affected Limb	Lameness Grade (AAEP Scale)
z1	8 y	Thoroughbred	Fetlock joint OA	RF	4/5
z2	4 y	Standardbred	Scapulo-humeral OA	LF	4/5
z3	23 y	Criollo	SDFT tendonitis	RH	3/5
z5	17 y	Warmblood	Proximal interphalangeal joint OA	RH	3/5
z6	7 y	Warmblood	Centrotarsal joint OA	LH	3/5
z7	15 y	Pony	Medial femoro-tibial joint OA	RH	3/5
z8	12 y	Italian saddle horse	DDFT tendonitis-navicular disease	RF	3/5

OA = osteoarthritis; SDFT = superficial digital flexor tendon; DDFT = deep digital flexor tendon.

## Data Availability

Data is available under request to the authors.

## Supplementary Materials

The following supporting information can be downloaded at: https://www.mdpi.com/article/10.3390/vetsci9090456/s1.
