# Supplementary material for: Fetlock Joint Angle Pattern and Range of Motion Quantification Using Two Synchronized Wearable Inertial Sensors per Limb in Sound Horses and Horses with Single Limb Naturally Occurring Lameness
Source: Vet Sci. 2022 Aug 25;9(9):456. doi: 10.3390/vetsci9090456 (PMC9502055; doi:10.3390/vetsci9090456)
Supplement: Supplementary file 1 [file vetsci-09-00456-s001.zip › vetsci-1816852-supplementary.pdf]

```
clc
clear all
close all

%% General defines

fs_Movit= 200;  %% Sampling freq IMU
fs_OMC = 60;  %% Sampling freq OMC
ft_angle_peaks = 3; % cutoff frequency for minimum selection
ft_angle = 10;  % cutoff frequency for data smoothing

order = 4; % butterworth filter order

%% IMPORT FILE

% IMPORT IMU FILE

[Movit] = import_anatomical_angle_cavalli();

% IMPORT OCM FILE

[OMC] = import_OMC_angle();


%% RESAMPLE
% Resampling of the OMC data from 60 Hz to 200 Hz

Fsa = fs_OMC;
Fsd = fs_Movit;  % Desired Sampling Frequency

OMC_TIME = OMC.TIME(1,1):1/Fsd:OMC.TIME(end,1);
OMC_Angle_resampled = spline(OMC.TIME, OMC.ANGLE(:,1), OMC_TIME);

%% LOW PASS FILTER (10 HZ)

[B_low,A_low]=butter(order,ft_angle/(fs_Movit/2),'low');
OMC_filt = filtfilt(B_low,A_low,OMC_Angle_resampled);
MOVIT_filt = filtfilt(B_low,A_low,MOVIT.ANGLE);

%% LOW PASS FILTER (3 HZ)
% signal used to find the first two maximum value among which to search the
```

```
% absolute minimum point (point used to sync IMU and OMC data in time and to
remove
% the offset between the OMC and IMU data)

[B_low_angle, A_low_angle] = butter(order, ft_angle_peaks/(fs_Movit/2),'low');
OMC_Angle_filt_3 = filtfilt(B_low_angle,A_low_angle,OMC_filt);
MOVIT_Angle_filt_3 = filtfilt(B_low_angle,A_low_angle,MOVIT_filt);

%% SESSION CUT

% from the data, the user select the start and the end of the
% valid session, removing tha recording parts to not consider into analysis

figure()
plot(OMC_TIME, OMC_Angle_resampled)
hold on
plot(MOVIT.TIME, MOVIT.ANGLE)
A = ginput(2)
legend('OMC', 'MOVIT')

Start_Point = A(1);
Stop_Point = A(2);

Movit_start_interval = find(MOVIT.TIME>= Start_Point, 1);
Omc_start_interval = find(OMC_TIME>= Start_Point, 1);

Movit_stop_interval = find(MOVIT.TIME>= Stop_Point, 1);
Omc_stop_interval = find(OMC_TIME>= Stop_Point, 1);

% cut 10 hz signal

MOVIT_Angle_selection = MOVIT_filt(Movit_start_interval: Movit_stop_interval);
MOVIT_Time_selection = MOVIT.TIME(Movit_start_interval: Movit_stop_interval);

OMC_Angle_selection = OMC_filt(Omc_start_interval: Omc_stop_interval);
OMC_Time_selection = OMC_TIME(Omc_start_interval: Omc_stop_interval);

% cut 3 hz signal
OMC_Angle_filt_3_selection = OMC_Angle_filt_3(Omc_start_interval:
Omc_stop_interval);
MOVIT_Angle_filt_3_selection = MOVIT_Angle_filt_3(Movit_start_interval:
Movit_stop_interval);
```

```
%% In the 3 hz filtered signal, find the first two maximum value

[ OMC_first_max, OMC_second_max ] = findFirstAndSecondMax( OMC_Time_selection,
OMC_Angle_filt_3_selection );
[ MOVIT_first_max, MOVIT_second_max ] = findFirstAndSecondMax(
MOVIT_Time_selection, MOVIT_Angle_filt_3_selection );


%% knowing the first two maximum value in time, find the absolute minimum value
between the two maximum in the 10 hz filtered data
[ OMC_t_min, OMC_value_min ] = findAbsoluteMinPoint( OMC_first_max,
OMC_second_max, OMC_Angle_selection, OMC_Time_selection);
[ MOVIT_t_min, MOVIT_value_min ] = findAbsoluteMinPoint( MOVIT_first_max,
MOVIT_second_max, MOVIT_Angle_selection, MOVIT_Time_selection);

% delta time and delta value between the minimum of the OMC signal and the
% minimum of the IMU signal

DELTA_TIME = OMC_t_min - MOVIT_t_min ;
DELTA_VALUE = OMC_value_min - MOVIT_value_min;

% remove time and value offset in the OMC signal
OMC_Time_selection_delta = OMC_Time_selection - DELTA_TIME;
OMC_Angle_selection_delta = OMC_Angle_selection - DELTA_VALUE;

%% After the temporal alignment, cut the signal and resample the OMC signal in order
to have the same number of samples to perform RMSE and Pearson coefficient
calculation

TIME_START = max(OMC_Time_selection_delta(1) , MOVIT_Time_selection(1));
TIME_STOP = min(OMC_Time_selection_delta(end) , MOVIT_Time_selection(end));

Movit_time_start_sample = find(MOVIT_Time_selection>= TIME_START, 1);
Movit_time_stop_sample = find(MOVIT_Time_selection>= TIME_STOP, 1);

t_resample =
MOVIT_Time_selection(Movit_time_start_sample:Movit_time_stop_sample);
OMC_signal = spline(OMC_Time_selection_delta, OMC_Angle_selection_delta,
t_resample);
MOVIT_signal =
MOVIT_Angle_selection(Movit_time_start_sample:Movit_time_stop_sample);
```

```
%% CALCOLO PEARSON E RMSE

for i = 1:length(t_resample)
	error(i) = OMC_signal(i) - MOVIT_signal(i);
end
RMSE = sqrt(mean(error.^2))

Corr = corrcoef([OMC_signal, MOVIT_signal]);
Pearson = Corr(1,2)
```
